# Common Mistakes in Managing Patients with Inflammatory Bowel Disease

**DOI:** 10.3390/jcm13164795

**Published:** 2024-08-14

**Authors:** Javier P. Gisbert, María Chaparro

**Affiliations:** Gastroenterology Unit, Hospital Universitario de La Princesa, Instituto de Investigación Sanitaria Princesa (IIS-Princesa), Universidad Autónoma de Madrid (UAM), Centro de Investigación Biomédica en Red de Enfermedades Hepáticas y Digestivas (CIBEREHD), 28006 Madrid, Spain; mariachs2005@gmail.com

**Keywords:** Crohn’s disease, ulcerative colitis, inflammatory bowel disease, mistake, error, misconception

## Abstract

**Introduction:** Errors are very common in medical practice and in particular, in the healthcare of patients with inflammatory bowel disease (IBD); however, most of these can be prevented. **Aim:** To address common errors in the management of IBD. **Methods:** Our approach to this problem consists in identifying mistakes frequently observed in clinical practice (according to our experience) in the management of patients with IBD, then reviewing the scientific evidence available on the subject, and finally proposing the most appropriate recommendation for each case. **Results:** The most common mistakes in the management of IBD include those related to diagnosis and differential diagnosis, prevention, nutrition and diet, treatment with different drugs (mainly 5-aminosalicylates, corticosteroids, thiopurines, and anti-TNF agents), extraintestinal manifestations, anemia, elderly patients, pregnancy, and surgery. **Conclusions:** Despite the availability of guidelines for both disease management and preventive aspects of IBD care, a considerable variation in clinical practice still remains. In this review, we have identified common mistakes in the management of patients with IBD in clinical practice. There is a clear need for a greater dissemination of clinical practice guidelines among gastroenterologists and for the implementation of ongoing training activities supported by scientific societies. Finally, it is desirable to follow IBD patients in specialized units, which would undoubtedly be associated with higher-quality healthcare and a lower likelihood of errors in managing these patients.

## 1. Introduction

In daily clinical practice, constant decision making is a necessity, and each decision is susceptible to potential errors [1,2,3,4,5]. Mistakes are very common in clinical practice, but importantly, most of them can be prevented. Over two decades ago, the Institute of Medicine released a groundbreaking report titled “To Err is Human: Building a Safer Health System”, revealing that approximately 100,000 Americans die annually from preventable errors in hospitals [6]. This publication significantly altered the discourse on healthcare quality, reshaping perceptions of care quality, garnering interest from payers and employers in enhanced care and patient safety, and prompting substantial increases in research support [5].

It is not our intention to address the topic of safety or the analysis of types of errors, although every doctor should be familiar with the fundamental literature on this subject [1,2,3,4,5]. However, it is worth recognizing that a good review of knowledge can have an impact (prevention) on certain human errors. Some errors (rule-based mistakes) arise from automatically applying a learned rule, either because it is not appropriate in a specific situation or because it is fundamentally flawed. The other type of error can stem from simple ignorance. In fact, historically, medical errors have often been attributed to unawareness. However, the rapid expansion of knowledge poses a new challenge: information overload.

It is acknowledged that some degree of variation is inherent in medicine due to its dual nature as both art and science [7]. However, in many instances, current care processes surpass the anticipated levels of natural variation, potentially indicating suboptimal overall care [7]. When faced with the same set of facts, different healthcare providers often make different diagnoses and prescribe different therapies [7,8,9]. The wide variations in practice may stem from the need for more evidence, the possibility of multiple equally effective approaches, or the insufficient consolidation and dissemination of existing evidence through guidelines [10]. Despite the widespread availability of guidelines and protocols, marked variation persists across all medical fields, reflecting poor quality of care [7,11,12].

In gastroenterology, inflammatory bowel disease (IBD)—including both Crohn’s disease (CD) and ulcerative colitis (UC)—is particularly prone to deviations from guidelines and notable variations in care processes [10,13,14,15,16,17,18,19]. There are at least two factors that establish IBD as a target for variation: (1) the presentation of IBD is heterogeneous, and the multiple presentations of the disease mandate different diagnostic and therapeutic approaches; and (2) the treatments for IBD are varied, and new treatments are always being developed and disseminated [7]. Significant variations in IBD care create a need for better information dissemination, and identifying factors predicting extremes in resource utilization may aid in targeting areas requiring improved knowledge or additional education [7].

While, in general, a consensus exists on diagnostic decision making in IBD, substantial variation persists in therapeutic decisions [7]. Specialist IBD clinics generally provide superior care, but even in these settings, a relevant minority of patients may not meet certain criteria [20]. Vignette surveys measuring decision-making variations highlight disparities between community gastroenterologists and IBD experts, emphasizing the need for further investigations into practice patterns [10]. Additionally, some studies reveal that patients with IBD who are referred for a second opinion often have not received previous or optimal medical therapy [21,22]. In a study recently conducted by our group, we aimed to identify the most common errors in the outpatient management of patients with IBD [22]. Consecutive patients diagnosed with IBD who were seen for a second opinion in our IBD Unit were included. Data on the strategies employed by physicians who had previously treated them were obtained and compared with currently recommended diagnostic and therapeutic procedures/guidelines. This study demonstrates that errors in the management of IBD patients are very frequent, both among general gastroenterologists but also among IBD specialists [22].

Previous studies have suggested that there is often a gap between guidelines and clinical practice [23], as well as between patients’ and physicians’ perspectives [24]. Adherence to guidelines—for example, European Crohn’s and Colitis Organisation (ECCO) guidelines—appears to be suboptimal overall for both therapeutic care and preventive health aspects of chronic disease management [23].

The present review aims to address common errors (according to our experience) in IBD (Table 1). Our approach to the problem will consist in identifying mistakes frequently observed in clinical practice in the management of patients with IBD, then reviewing the scientific evidence available on the subject, and finally proposing the most appropriate recommendation for each case.

## 2. Diagnosis and Differential Diagnosis

### 2.1. When Admitting Previously Diagnosed UC Patients with Rectal Bleeding, It Is Not Necessary to Rule Out an Enteric Infection as It Is Evident that It Is a Flare-Up of Their IBD

Intestinal infections can mimic the clinical and even endoscopic manifestations of UC [25]. Additionally, various infectious agents can cause superinfection in patients with IBD, which can trigger a relapse or worsen a flare-up of this disease [26]. Therefore, in every patient admitted for a presumed flare of UC, infectious causes of diarrhea should be excluded [27]. Thus, even if the diagnosis of UC is already known, it is advisable to request stool cultures to rule out a possible infection by *Salmonella*, *Shigella*, *Campylobacter*, or *Escherichia coli* [27]; *Clostridioides difficile* (*C. difficile*) infection, in particular, is addressed in the following section.

However, the actual yield of routine pathogen determination in stool in clinical practice for patients with a supposed IBD relapse is not clearly established and appears to be relatively low. A recent trial in which systematic stool studies were conducted in patients presenting with an IBD flare showed that stool cultures were positive in only 4% of cases [28]. More recently, other researchers have confirmed these disappointing results by prospectively evaluating over 2 years the incidence of intestinal superinfection by enteropathogens (through systematic stool cultures) in 99 IBD flare-ups requiring hospitalization; this diagnostic strategy detected only five bacterial infections (by *C. jejuni*), representing just 5% of cases, and no correlation between the infection and the course of the disease could be demonstrated [29].

However, it is important to highlight that while collectively the number of patients with supposed IBD relapse and concomitant bacterial infection seems low, the consequence of correctly identifying such infections is very significant at the individual level. Furthermore, the increasing use of drugs that significantly modify the response to infections in IBD (immunomodulators, biological agents, and small molecules) may lead to a higher frequency of infectious complications.

It has been suggested that symptoms and signs (such as a high fever and marked leukocytosis) may sometimes lead a clinician to suspect infection as a cause of an IBD flare-up. Nevertheless, although it can be difficult or even impossible to differentiate between an IBD flare and enteroinvasive diarrhea, steroid treatment should not be delayed while awaiting stool culture results (which may take several days) in patients with a previously established UC diagnosis or a well-founded suspicion of this disease [27].

### 2.2. C. difficile Infection Should Only Be Considered in IBD Patients Who Have Recently Received Antibiotics

*C. difficile* infection has been associated with IBD exacerbations [30]. It is generally considered that every patient admitted for a severe IBD flare should be systematically examined for *C. difficile* toxin in their stools, regardless of whether they have previously received antibiotic treatment [30]. IBD is an independent risk factor for *C. difficile* infection, even in the absence of traditional risk factors such as antibiotic exposure and hospitalization [31,32]; additionally, patients do not always accurately remember if they have taken antibiotics, particularly if this occurred several weeks before the onset of colitis. A meta-analysis including 12 studies reported a significant association between community-acquired *C. difficile* infection and IBD [33]. A population-based study revealed that patients with IBD were approximately five times more likely to develop *C. difficile* than patients without IBD [34]. Furthermore, *C. difficile* is significantly more frequent in IBD patients experiencing flares than in those with inactive IBD [35]. In fact, *C. difficile* negatively impacts short- and long-term IBD-related outcomes, including hospitalization, colectomy, and mortality rates [36,37,38]. Finally, regarding the effect of *C. difficile* infection treatment on IBD activity, some studies have shown a beneficial effect of antibiotic treatment on IBD [39].

Accordingly, the ECCO guidelines on the prevention, diagnosis, and management of infections in IBD concludes that “screening for *C. difficile* infection is recommended at every disease flare in patients with IBD and especially in patients receiving immunosuppressive therapy” [40]. In the same way, the guideline for diagnostic assessment in IBD jointly organized by the ECCO and the European Society of Gastrointestinal and Abdominal Radiology (ESGAR) concludes that “all patients with a suspected new flare of IBD should be investigated for infection, including exclusion of *C. difficile* infection” [41].

### 2.3. Assume That All Cases of Proctitis Are Ulcerative Proctitis

The increase in the incidence of IBD and the growing awareness among healthcare professionals about this problem have led to improved diagnosis for these patients. However, we must not forget that other diseases can present with manifestations that mimic IBD [42,43]. In this regard, sexually transmitted infections can manifest with symptoms, endoscopic findings, and histological features that overlap with those of IBD, posing a challenge in terms of differential diagnosis [44]. In fact, the endoscopic appearance (inflammation or ulcers) and histologic changes (acute inflammation) in the mucosa in infective and inflammatory colitis may be nearly indistinguishable [45,46]. Other conditions that typically mimic IBD include ischemic colitis and diverticular colitis.

In recent years, the incidence of proctocolitis due to sexually transmitted infections has increased, especially in individuals with high-risk sexual practices [47]. The most frequently implicated microorganisms are *Neisseria gonorrhoeae*, *Chlamydia trachomatis* (causing lymphogranuloma venereum), *Treponema pallidum*, and the monkeypox virus, which caused a pandemic outbreak in 2022.

Almost a third of patients presenting with mucoid bloody diarrhea and suspected IBD have an infective etiology, and in addition, patients with IBD are prone to bacterial superinfection [48]. The most common enteric pathogens involved are *Campylobacter*, *Salmonella*, *Shigella*, *Amoeba*, and *C. difficile*.

In a patient diagnosed with IBD who presents with any atypical clinical, endoscopic, or histological feature or a lack of response to treatment, it is recommended to rule out other diagnoses. A medical history that addresses their intestinal and extraintestinal symptoms, travels, and sexual behavior (unprotected passive anal intercourse) is required. To reach a definitive diagnosis, thorough endoscopic and histological evaluation of the lesions is necessary, and microbiological tests and serologies are usually also needed.

### 2.4. The Endoscopic Lesions of UC Are Always Continuous

No endoscopic feature is specific for CD or UC. The most useful endoscopic features of UC are considered to be continuous, and there is confluent colonic involvement with a clear demarcation of inflammation and rectal involvement [41]. However, although the endoscopic involvement of UC typically begins in the rectum and extends continuously, more or less proximally, several series have described cases of discontinuous or segmental lesions. For example, the involvement of the right colon (or the inflammation of the periappendicular area) has been observed in patients with left-sided colitis, or some patients may even have an intact rectum [49,50,51,52,53,54,55,56,57,58,59,60]. In fact, any segment is susceptible to containing segmental lesions, but the periappendicular area and the cecum appear to be the most likely sites for these discontinuous lesions [59]. While rectal sparing or patchy involvement typically raises suspicions of CD, it does not appear that these patients are subsequently diagnosed more frequently with CD [60,61]. Thus, the presence of segmental lesions alone does not exclude a UC diagnosis.

In any case, during the first exploration (without previous medical treatments), the initial assessment of the disease extent should be very detailed to facilitate diagnosis and classification. After the administration of topical treatment, there may be a disproportionate improvement, or even apparent normality, of the distal colonic segments (especially the rectum) compared to the more proximal area [50,51,62]. This may be misinterpreted as segmental colonic involvement. It has also been suggested that systemic treatment (oral or intravenous) may also be responsible for the non-uniform improvement of colonic mucosa, resulting in the more evident disappearance of lesions in some portions of the colon than others [50,63].

### 2.5. In Severe UC Flare-Ups, a Complete Colonoscopy Is Necessary to Precisely Define the Extent of the Disease and Choose the Most Appropriate Treatment

Firstly, it is important to emphasize that in a severe UC flare-up (in a patient with an already established diagnosis), detailed knowledge of the extent of the disease will not change the therapeutic approach, as intravenous steroid treatment (or in some cases, biological agents or small molecules) will be necessary regardless of the length of colonic involvement. Secondly, it should be noted that a colonoscopy could trigger a toxic megacolon in these severely ill patients [27], probably due to the distension caused by the procedure, which in turn affects the blood supply to the colon wall, increasing the mucosal uptake of bacterial products [64]. Therefore, complete colonoscopies should be avoided as much as possible in severe cases of UC (or CD). Consequently, if endoscopic examination of the colon is necessary, it should be as limited as possible—exploring only the rectum and, at most, the most distal part of the sigmoid colon—and carried out with utmost care, inflating the least amount of air possible and aspirating at the end of the procedure [41,63]. This brief and limited endoscopic examination will be sufficient to assess the severity of the lesions (keep in mind that in UC, the most apparent involvement is usually found in the most distal segment) and to obtain biopsies from the rectum to assess the patient for cytomegalovirus (CMV) infection.

### 2.6. An Obstructive Picture in Patients with CD Is Always Due to Intestinal Stenosis as a Consequence of Their Underlying Disease

Intestinal stenosis due to CD produces a series of symptoms typical of any intestinal obstructive process: colicky pain, abdominal distension, vomiting, and the worsening of symptoms after food intake. Although the onset of symptoms of a partial obstruction can be abrupt, stenotic CD almost never presents in a “catastrophic” manner as a total obstruction, and in most cases, there is no associated vascular involvement [65]. Therefore, the identification of a complete obstruction should suggest another cause, such as adhesions secondary to a previous surgery, a hernia, or an intestinal volvulus. It is essential to distinguish strangulation due to these latter complications, which require immediate surgery, from an obstruction secondary to luminal narrowing caused by CD [65]. Initially, this differentiation is often impossible since there are no pathognomonic signs of strangulation, although its clinical presentation is usually more severe. Therefore, strict observation, with the monitoring of clinical, radiological, and laboratory evolution, is of paramount importance. Finally, it should be noted that an uncomplicated intestinal obstruction due to CD almost invariably resolves spontaneously (or with medical treatment) and rapidly (i.e., within two or three days), so the absence of evident improvement within this timeframe should raise suspicion of another diagnosis and prompt consideration of surgical treatment [13].

### 2.7. The Clinical Manifestations of Toxic Megacolon Are Very Characteristic, So Its Diagnosis Is Usually Straightforward

The characteristic clinical features of toxic megacolon typically include bloody diarrhea refractory to medical treatment for a week or more (although sometimes the onset of symptoms can be faster) [64]. In patients with UC, unlike in CD, continuous abdominal pain is rare (although colicky pain relieved by defecation is common), so its presence, in a patient with severe UC, suggests the possibility of a toxic megacolon [66]. Additionally, since severe abdominal pain is not typical in a UC flare, its appearance might suggest the possibility of colonic perforation [66].

However, diagnosing a toxic megacolon is not always straightforward. Although diarrhea is almost always initially present, later it may be decreased or even evolve into constipation, probably due to the loss of colonic motility (referred to as a “false improvement”) [67]. Accordingly, physical examinations typically reveal decreased or absent bowel sounds and increased abdominal tympanism [67]. Finally, steroid treatment may mask the symptoms and signs of toxic megacolon, making early diagnosis even more challenging [67]. Therefore, to accurately diagnose this condition, it is necessary to initially perform a plain abdominal X-ray in every patient with a severe IBD flare, regardless of whether they present abdominal pain or diarrhea, and subsequently and frequently (e.g., every 24 or 48 h) during the follow-up of the flare, mainly in the case of no observed improvement.

### 2.8. CMV Infection, Whenever Present, Always Plays a Causative Role in the Flare-Up of UC or in the Episode of Corticosteroid Refractoriness

Numerous studies have been published evaluating the role of CMV infection in patients with IBD [68]. Additionally, several studies have suggested an etiological role of this infection, especially in corticosteroid-refractory UC [68]. Furthermore, adequate responses to antiviral therapy (e.g., ganciclovir) have been repeatedly described in corticosteroid-refractory UC [69]. However, in some cases, antiviral treatment has failed to induce clinical remission or prevent surgery [69]. Moreover, clinical improvements have been observed in some patients with UC with concomitant CMV infection who received only steroid treatments (without antiviral therapy), indicating that, at least in some instances, this microorganism may merely act as a commensal or “innocent bystander” [70]. The practical recommendation arising from this conclusion is that, regardless of the decision to administer treatment against CMV, the underlying disease (UC) should not be left untreated.

## 3. Prevention

### 3.1. For Patients with CD Who Smoke, Repeatedly Emphasizing the Necessity of Quitting Smoking May Not Be So Crucial

Despite the known association between smoking and the increased need for steroids, immunosuppressants, and surgery for CD and the improvements in disease course that occur with smoking cessation [71,72], not all smokers with CD receive proper counseling [23]. Although counseling regarding smoking cessation appears to have improved compared with previous studies [73,74], there is still room for improvement. It is imperative that gastroenterologists familiarize themselves with the resources and strategies available for smoking cessation [75], as unassisted attempts to quit have been associated with a low chance of success, with only 3–5% of such attempts resulting in long-term abstinence [71].

### 3.2. Early Screening for Latent Tuberculosis Is Not Necessary; It Is Sufficient to Screen Only When the Patient Already Requires Immunosuppressive Treatment

We understand that “early” screening for latent tuberculosis is conducted when there is still no indication for biological (or small-molecule) therapy, and ideally, it should be performed at the diagnosis of IBD, before the patient receives immunosuppression, and preferably with a low inflammatory burden [76]. Often, at diagnosis, there is a high inflammatory burden that makes early screening impossible. Therefore, in these cases, screening should be performed at a later period when the patient is in a state of immune competence. Early latent tuberculosis infection screening is recommended for all IBD patients since all of them could potentially require future treatment with biological agents or small molecules [40,77]. The SEGURTB study has shown that the likelihood of a positive result in the early tuberculin skin test (performed before an indication for biological therapy but not necessarily at diagnosis, without associated immunosuppression and with a low inflammatory burden) is double that of the mandatory tuberculin skin test performed right before starting an anti-TNF [76].

In the case of a positive early screening test, it is advisable to delay tuberculosis chemoprophylaxis until the patient receives biological treatment or small molecules [76]; we must avoid over-treating (with isoniazid) patients who will not need treatment with biological agents or small molecules. Moreover, early chemoprophylaxis would not protect against new contacts with *M. tuberculosis* that may occur in the interval between the positive early test and the start of biological/small molecule therapy. It is essential to explain this recommendation to the patient, considering that although performing chemoprophylaxis for a positive early screening test is not considered strictly incorrect, it does not seem justified from an epidemiological perspective [77].

### 3.3. Routinely Assessing the Need for Vaccination at the Time of Diagnosis Is Not Necessary in Patients with IBD

Patients with IBD are at an increased risk of infection, in part owing to the disease itself, but mostly because of treatment with immunosuppressive drugs [78], and they are likely to need immunosuppressive therapy during the course of their disease.

The fatality rate of fulminant hepatitis A virus (HAV) infection has been estimated to be up to 2% in adults over 40 years, and a higher rate is suggested in immunosuppressed patients [40]. The reactivation of hepatitis B virus (HBV) is a well-known complication of immunosuppression [79,80]. The risk of CMV reactivation is increased in IBD patients exposed to corticosteroids or thiopurines but not in those treated with anti-TNF agents [81]. CMV-seropositive patients receiving immunosuppressors are at risk of virus reactivation, whereas seronegative patients acquire primary CMV infection infrequently [40]. Primary Epstein–Barr virus (EBV) infection in EBV-negative patients appears to be a risk factor for lymphoproliferative disease [82]. Thus, the ECCO guidelines on the prevention, diagnosis, and management of infections in IBD states that “serological screening for hepatitis A, B, C, HIV, EBV, CMV, varicella zoster virus, and measles virus (in the absence of documented past infection or vaccination for the latter two) is recommended for all IBD patients at baseline” [40]. Furthermore, some vaccines, particularly live-agent vaccines, cannot be safely administered to immunocompromised IBD patients. Additionally, the response rate to certain vaccines, such as hepatitis B, is often low in IBD patients on immunosuppressors or anti-TNF therapy [83,84,85]. All of this underscores the recommendation that the optimal time for immunization is at diagnosis, before starting any immunosuppressive treatment.

Thus, the current method for preventing opportunistic infections in IBD patients involves a comprehensive clinical and laboratory work-up (the use of standardized checklists may be useful) before beginning treatment with immunosuppressors/biological therapies/small molecules, with catch-up vaccinations for incomplete series [86]. The vaccination schedule should also include combined vaccinations against tetanus, diphtheria, and inactivated poliomyelitis every 10 years, annual influenza vaccination, and pneumococcal vaccination every 5 years.

In spite of these guidelines, vaccines are underutilized in IBD patients, indicating a gap in immunization against vaccine-preventable illnesses despite significant risk factors [87,88,89,90,91]. In some studies, the primary reason for non-immunization was that the vaccine was not offered, highlighting the need for better patient education by healthcare providers and also the need for education among healthcare professionals [92]. In this respect, gastroenterologists’ knowledge of appropriate immunizations for IBD patients is clearly deficient [88].

## 4. Nutrition and Diet

### 4.1. Self-Imposed Food Restrictions Help Prevent the Onset of IBD Flare-Ups and Aid in Controlling Their Activity

In IBD patients, reduced oral intake can significantly contribute to the onset of malnutrition. This reduction can result from various factors, including self-imposed food restrictions, decreased hunger, diminished pleasure from eating, mood changes, and even medical advice [93,94]. In a prospective, multicenter study including 1271 IBD patients from outpatient clinics, a questionnaire was applied to obtain data on the patients’ dietary behavior and beliefs [95]. The vast majority of the IBD patients had self-imposed food restriction behaviors in order to prevent a disease flare and because of fear of worsening disease symptoms during a flare [95]. Other studies have also described a high prevalence of self-reported food avoidance and restrictive dietary behavior in patients with IBD [95,96,97,98,99]. Many beliefs of the patients could be perpetuated by professional dietary advice [93,100]. However, scientific evidence to support specific dietary advice in patients with IBD is currently lacking [101], while dietary restrictions predispose individuals with IBD to nutrition-related complications and have a negative psychosocial influence.

### 4.2. Patients Admitted for an IBD Flare Benefit from Complete Fasting, as It Reduces Disease Activity; The Administration Route for Nutritional Supplements Should Be Parenteral, as It Is More Effective and Better Tolerated Than Enteral Feeding

Malnutrition is frequently associated with IBD during active phases [102]. Furthermore, patients admitted for a severe flare of IBD are often malnourished, in a catabolic state, and will also be required to fast for the frequent tests they undergo. However, neither total parenteral nutrition nor absolute fasting have proven effective in treating IBD [103,104,105]. González-Huix et al. compared the role of total enteral and parenteral nutrition as adjunctive therapy to steroids in severe UC, and found that while they were equivalent, the former was more cost-effective and associated with fewer adverse effects [105]. This is not surprising as enteral nutrition is more physiological and lacks the complications associated with parenteral nutrition, such as catheter-related infections or various metabolic issues [106].

Maintaining a patient with UC (even if severe) on absolute fasting in an attempt to achieve “intestinal rest” is not only not beneficial but worsens their malnutrition status and can be harmful. Absolute fasting deprives colonocytes of contact with short-chain fatty acids, vital for their metabolism and repair [107]. Only in cases of IBD complicated by intestinal obstruction, massive bleeding, a toxic megacolon, or suspected perforation should fasting and total parenteral nutrition be considered. Conversely, patients with IBD (mainly hospitalized ones) should be evaluated for the need for artificial nutrition, typically through oral supplements. In summary, if nutritional support is required, enteral nutrition should be the preferred alternative, a recommendation reflected in the aphorism stating “when the intestine works, use it” [108].

## 5. Aminosalicylates

### 5.1. Aminosalicylates Are Equally Effective for Treating CD and UC

Aminosalicylates (5-ASA) are unequivocally regarded as the primary choice for both treating and sustaining remission in UC [109,110,111]. However, the role of 5-ASA in the management of CD has been a subject of controversy [112,113,114]. An initial meta-analysis of three placebo-controlled trials of Pentasa^®^ in patients with active CD showed a mean reduction of 63 points in the Crohn’s Disease Activity Index (CDAI), compared to a 45–point reduction with a placebo (a small difference of only 18 points) [115]. More recently, the ECCO working group conducted a meta-analysis of seven randomized controlled trials comparing induction therapy with oral mesalazine or sulfasalazine versus a placebo in patients with active CD [116]. The results showed similar clinical remission rates between 5-ASA therapy and a placebo, consistent with findings of a meta-analysis conducted by the Cochrane collaboration [117]. Additionally, adverse-event-related treatment withdrawals were comparable between the treatment and placebo groups. Accordingly, the ECCO guidelines recommend against the use of 5-ASA for the induction of CD remission [116]. Finally, it should be mentioned that one published network meta-analysis noted a small statistically significant effect on clinical remission among the study arms which evaluated 5-ASA at daily doses of >2.4 g/day [118]; however, another network meta-analysis was unable to confirm this dose effect [119].

Regarding maintenance treatment, initially, a Cochrane systematic review found no evidence to suggest that oral 5-ASA preparations are superior to a placebo for the maintenance of medically induced remission in patients with CD [120]. In total, 11 placebo-controlled clinical trials evaluated doses ranging from one to four g per day [120]. Treatment durations varied from 4 to 36 months, with a 12-month evaluation being the most common. No statistically significant benefit was found for clinical outcomes with oral 5-ASA. No benefit was observed based on disease location, including in patients with colonic-only involvement. Accordingly, the ECCO guidelines recommend against the use of oral 5-ASA as maintenance therapy in CD [116]. Finally, while there have been some suggestions of benefits for maintaining remission in small-bowel CD after surgical resection, the effect sizes are, in any case, very small [121,122].

A survey featuring five vignettes to gather provider beliefs about the appropriateness of therapies for CD has assessed the level of agreement between community gastroenterologists and IBD experts, with the latter presumably adhering more closely to practice guidelines [7]. For managing a patient with newly diagnosed CD, 75% of community providers recommended the use of 5-ASA products, compared to less than half (44%) of the experts [7].

### 5.2. The Combination of Oral and Topical Aminosalicylates Is Deemed Unnecessary, as Each Treatment Alone Demonstrates Similar Efficacy

Pharmacokinetic research indicates that orally administered 5-ASA primarily targets the distal ileum and proximal large bowel, resulting in a higher concentration of the active compound in the right colon compared to the left colon. On the contrary, only minimal quantities of the drug are found in the rectal mucosa [123,124]. Conversely, when 5-ASA is administered topically, it ensures significant drug availability in the rectosigmoid sites and, to a lesser extent, in the descending colon [125,126]. Thus, it seems that to enhance the mucosal 5-ASA concentration throughout the entire length of the large bowel in UC patients, besides oral dosage, topical treatment should be considered [111,127,128]. In fact, in left-sided UC (and in ulcerative proctitis), the efficacy profile of topical 5-ASA is superior to oral 5-ASA therapy (and to topical steroids) [129].

Only a few trials, including 322 patients and with a treatment duration of 3–8 weeks, have compared the use of oral 5-ASA combined with topical 5-ASA versus oral 5-ASA as a monotherapy for the induction of remission in patients with active UC [111]. In all of these studies, the desirable effects of 5-ASA combined therapy (compared with oral monotherapy) probably outweigh the undesirable effects of this intervention, although the level of uncertainty is high [130,131,132,133]. Moreover, another study reported that combined oral and topical mesalazine treatment significantly improved health-related quality of life in patients with active UC [134]. Two trials compared these two therapeutic strategies for clinical response in patients with disease of at least a rectosigmoid extent [130,132]. In the pooled analysis, no significant advantage of combined therapy over 5-ASA monotherapy in clinical response was observed [111]; however, these trials were heterogeneous in terms of study design, 5-ASA doses, the definition of clinical activity, and the definition of clinical improvement [111]. The only trial comparing combined versus oral 5-ASA therapy on endoscopic activity of UC showed a higher endoscopic remission rate with the combined regimen [132]; however, the difference was not statistically significant [111]. Finally, combined oral and topical 5-ASA therapy also seems to exhibit a favorable cost–benefit ratio in pharmacoeconomic analyses [135,136].

Based on the aforementioned data, the ECCO guidelines on medical treatment of UC suggest the use of oral 5-ASA (≥2 g/d) combined with topical (rectal) 5-ASA over oral 5-ASA monotherapy for the induction of remission in adult patients with active UC of at least a rectosigmoid extent. While many authors have asserted that patients generally find long-term rectal treatment acceptable, a postal survey of British patients revealed that 80% preferred oral treatment alone [137]. Therefore, this form of combination treatment (aimed at maintaining remission) could be reserved for patients with a high likelihood of relapse [138]. Consequently, adding rectal therapy becomes a viable treatment option for patients who have experienced a relapse while on oral 5-ASA alone.

### 5.3. The Total Dose of Aminosalicylates Should Be Split into at Least Two Daily Administrations, as a Single Daily Dose Is Less Effective

Multiple-dose daily regimens can disrupt patients’ normal daily activities and diminish their overall quality of life, leading to decreased treatment adherence and potentially worse long-term outcomes [139]. UC colitis patients often identify the treatment regimen’s complexity, the number of tablets, and the frequency of doses as significant barriers to their adherence [139,140].

Pharmacokinetic studies conducted in healthy volunteers have indicated that once-daily dosing could be a viable option for patients with UC [141,142]. The response to 5-ASA is closely associated with tissue concentrations and is best anticipated by examining drug concentrations within the colon lumen. Some researchers have utilized computer simulations, supporting the notion of the once-daily administration of 5-ASA as the standard treatment for UC [143]. In fact, several meta-analyses have demonstrated that once-daily dosing with 5-ASA is as effective and safe as conventional dosing schedules both for induction and for maintenance treatment in UC [144,145,146,147,148]. Furthermore, some studies have reported that patients with UC who receive 5-ASA once daily demonstrate superior remission rates, levels of acceptability, and self-reported adherence to therapy compared to those given 5-ASA twice daily [149].

The collective evidence indicates that the effectiveness of once-daily dosing for all these compounds might be attributed to the pharmacodynamic properties of 5-ASA, rather than to the specific characteristics of the formulation determining drug delivery. In other words, the effect is likely to be generic rather than specific to a particular 5-ASA compound.

## 6. Corticosteroids

### 6.1. Corticosteroids Are Generally Used Appropriately (Only When Necessary)

Corticosteroids remain, at present, one of the most useful group of drugs for treating acute IBD flares. Their high potency and low cost are only offset by their significant side effects, especially if their use is prolonged [150,151]. Thus, despite the development and incorporation of new therapeutic strategies, such as biological agents and small molecules, corticosteroids still play an important role in inducing remission in patients with IBD.

A frequent and relevant mistake is to use corticosteroids as maintenance treatment, either because they are not discontinued or because they are often prescribed without a maintenance therapy strategy [151,152,153]. Various authors have suggested that the (lack of) use of corticosteroids should be a quality-of-care indicator for IBD programs [154,155,156]. Despite this, a significant percentage of patients are still inadequately treated with corticosteroids [150,151]. Some studies indicate that 30–50% of IBD patients are still being exposed to corticosteroids annually, with 10–20% exposed to excessive corticosteroids [157]. It is noteworthy that approximately half of these cases could potentially be avoided [158,159,160,161,162]. Indeed, the misuse of corticosteroids is probably one of the most common bases for malpractice suits in the treatment of IBD [13]. Thus, although there are contraindications for prolonged corticosteroid use, an analysis of US claims data revealed that 10–25% of UC patients had received corticosteroid treatment for over 3 months during the 12-month study period [163].

Suitable alternatives to corticosteroids should always be considered. The timely introduction of immunomodulators/biologic agents/small molecules is essential, as these therapies have demonstrated corticosteroid-sparing potential in IBD. However, timely escalation when a patient is either corticosteroid-refractory or -dependent is not performed in a significant proportion of cases, leading to inappropriate corticosteroid excess [150,151]. In this respect, when compared to immunomodulators and biological therapies, the prolonged use of corticosteroids remains the primary risk factor for increased morbidity and mortality in patients with IBD [164]. The use of anti-TNF agents and the presence of multidisciplinary IBD teams are both associated with reduced levels of inappropriate long-term corticosteroid use [165]. The chronic or repeated use of systemic corticosteroids, without attempting steroid-sparing strategies in patients with IBD, represents low-quality care [114], despite the relatively lower direct costs of the medication compared with the costs of advanced therapies with steroid-sparing and disease-modifying benefits [114].

In order to avoid common mistakes with corticosteroids, physicians need to educate and engage patients and general practitioners (and also gastroenterologists) regarding the proper role of corticosteroids in IBD treatment, including information about the potential short-term and long-term side effects of corticosteroids [150,151]. Corticosteroid-free remission should be a key therapeutic target [166].

### 6.2. Corticosteroids Are Effective in Patients Who Are Already Receiving Treatment with Immunomodulators or Biological Agents

A meta-analysis including more than 4000 patients suggested that the combination of corticosteroids and an anti-TNF would only increase morbidity due to the presence of adverse events [167]. In a recent study, after one course of steroids administered to IBD patients receiving immunosuppressive treatment, only 35% remained in remission without needing treatment escalation [161].

### 6.3. It Is Recommended to Start with Low or Intermediate Doses of Corticosteroids, and Only Use Full Doses if No Response Is Observed

The approach of starting with low doses of corticosteroids and increasing them if the desired response is not achieved, with the intention of reducing the incidence of adverse effects, lacks a scientific basis. Once the decision to administer steroids has been made, they should be prescribed at doses that have been shown to be effective, namely “full” doses. This assertion is based on several arguments as follows. (a) The cumulative dose of steroids received by patients who are prescribed full doses from the beginning is often lower than that received when the treatment starts with low doses and gradually increases them. This latter strategy is frequently associated with an incomplete clinical response, necessitating dose escalation and ultimately resulting in a longer duration and higher total dose of steroids. (b) It has been suggested (though not clearly proven) that the gradual use of increasing doses of steroids may promote the development of corticosteroid resistance or dependence. (c) Employing full doses of steroids from the beginning facilitates defining a flare as refractory, because if a high-dose treatment fails to elicit a response, we can classify the patient as corticosteroid-resistant without doubts about using a dose that was possibly insufficient.

However, the optimal steroid dose for treating IBD is not well established. One study compared three doses of prednisone (20, 40, and 60 mg/day) in patients with UC and demonstrated that the two higher doses are more effective than 20 mg/day [168]. Nonetheless, the limited sample size of this study, and the consequent reduced statistical power, did not allow for a determination of differences in efficacy between the 40 and 60 mg/day doses of prednisone [168]. A meta-regression analysis did not find any correlation between increased corticosteroid dosing and a reduction in colectomy rate in patients with severe UC and concluded that doses beyond 60 mg per day of methylprednisolone or equivalent should not be used [169]. Most clinicians use these drugs at doses ranging from 0.75 to 1 mg/kg/day of prednisone (or equivalent) [128]. In a meta-analysis of 24 cohort studies in patients with acute severe UC, the mean dose of intravenous methylprednisolone was 68 mg, ranging from 40 to 100 mg/day [169]. Administering doses exceeding 1 mg/kg/day of prednisone does not increase efficacy and, conversely, is associated with a higher incidence of adverse effects [170].

### 6.4. At Least 10 Days Must Pass before Considering a Patient with Severe UC Treated with Intravenous Corticosteroids as Corticosteroid-Refractory

The determination of the period from which steroid refractoriness is defined is crucial, as over time, especially in patients who do not clearly worsen but do not improve either, serious complications can develop, sometimes masked by steroid administration. Furthermore, the categorization of a patient with UC as corticoresistant should be followed by the consideration of rescue therapy, either with cyclosporine/infliximab or surgery [171]. Corticosteroid resistance has traditionally been defined in severely ill UC patients (hospitalized) receiving intravenous corticosteroids as the absence of response after 7–10 days [172]. However, more recently, it has been suggested that corticosteroid response should be evaluated earlier. Thus, some authors have suggested that 3–5 days of treatment might be sufficient, while others consider that 5 days, or perhaps between 5 and 7 days at most, could be considered a reasonable period to assess steroid treatment response [27]. In any case, it has been shown that prolonging steroid treatment for more than 7–10 days in corticosteroid-resistant patients does not increase therapeutic response and, on the contrary, is detrimental because it increases adverse effects and delays the administration of other potentially effective rescue treatments [173,174]. It seems appropriate here to recall the sensible aphorism that urges us not to be obstinate when it comes to saving a patient’s colon, but rather the patient themselves [175,176].

### 6.5. Faced with a Patient with Severe UC Resistant to Corticosteroids Who Has a CMV Infection and Has Started Antiviral Treatment, It Is Necessary to Immediately and Completely Discontinue the Steroids

When CMV infection is detected in the colonic biopsies of a patient with severe corticosteroid-resistant UC, the physician faces the difficult dilemma of whether to suspend (rapidly) the immunosuppressive treatment, which would favor the response of the infection to antivirals but could worsen the activity of UC due to the underlying inflammatory disease [70]. The recommendation to rapidly taper, albeit progressively, the dose of steroids and other immunosuppressors in patients with corticosteroid-resistant UC who have been diagnosed with CMV infection and have started treatment with ganciclovir is widely followed. However, the benefit of this approach has never been demonstrated and is not as obvious as it might seem. It could also be argued that steroids may be useful for controlling concomitant inflammation, and therefore, combined treatment with steroids and ganciclovir would allow for action simultaneously on inflammation and infection. In this regard, it is unknown whether corticosteroid resistance is permanent in this situation of superinfection, but some indirect data indicate that steroid refractoriness is reversible in both experimental and clinical conditions [177]. Moreover, we do not know to what extent treating CMV infection reverses the inflammatory component perpetuated by viral reactivation. Lastly, we must recall the previously mentioned recommendation that, regardless of the decision to administer treatment against CMV, the underlying disease should not be left untreated. Therefore, an immediate or excessively rapid withdrawal of steroids in this situation does not seem prudent.

### 6.6. Since Bone Loss Does Not Begin to Occur until Several Months after the Start of Corticosteroid Treatment, It Is Not Necessary to Initially Administer Prophylactic Therapy for Osteopenia

Osteoporosis is characterized by a decrease in bone mass accompanied by a deterioration of bone tissue architecture, leading to increased bone fragility and consequently an increased risk of fractures. Corticosteroids decrease the amount of absorbed calcium and increase the amount excreted in urine [178]. It has been estimated that between 25% and 50% of patients receiving prolonged steroid treatment will suffer bone fractures [179]. Moreover, IBD itself is a significant risk factor for the development of osteopenia and osteoporosis [180,181].

The use of steroids in IBD is undoubtedly one of the most determining factors in the occurrence of bone metabolism alterations in these patients [180,182]. It has been calculated that very low doses of steroids (even 2.5 mg/day) are capable of inducing bone mass loss. Notably, the rate of bone mass loss is highest during the first 6–12 months of steroid treatment, and the detrimental effects of these drugs can be identified by bone densitometry at as early as 6 months [182,183]. Thus, during the first year of treatment, up to 15% of bone mineral mass can be lost. Moreover, the increase in the incidence of bone fractures may manifest as early as 3 months after starting steroid treatment [182].

It has been suggested that short-term steroid administration (approximately only for one month) is not associated with a decrease in bone mineral density. However, most IBD patients will need to take these drugs for several months (the duration of the usual steroid tapering regimen), and it is precisely at the beginning when bone loss will be greatest (among other things, because initially higher doses of steroids are used). Finally, it should be noted that, unfortunately, upon the discontinuation of steroids after a certain period of administration, the bone mass usually does not return to pre-treatment levels [178,180].

Taken altogether, the aforementioned evidence indicates that prophylactic treatment of osteopenia should begin early [184]. A practical option is to administer calcium and vitamin D from the beginning to all IBD patients requiring steroid treatment, including low-bioavailability oral corticosteroids. However, a study involving 131 gastroenterologists showed that only 38% prescribed vitamin D and calcium in this patient group [185].

## 7. Thiopurines

### 7.1. It Is Advisable to Split the Dose of Thiopurines into Several Intakes to Facilitate Gastric Tolerance

Thiopurine drugs, namely azathioprine and mercaptopurine, have demonstrated effectiveness in maintaining remission in IBD, mainly in the context of corticodependence [111,113,116,186]. A debated aspect has been whether to administer the drug daily as a single dose or divided into several doses. Divided doses have been described as a limiting factor for treatment adherence, particularly in the case of chronic medication, such as thiopurine therapy, which supports the administration of the full dose in a single daily intake from the start of treatment, as splitting the dose has not been shown to have any advantage [140]. Dividing the dose should only be considered in patients who experience certain side effects (mainly digestive intolerance) [187].

### 7.2. In Patients Who Develop Digestive Intolerance to Azathioprine, Thiopurine Drugs Should Be Permanently Discontinued

Azathioprine intolerance remains a significant clinical issue in patients with IBD, leading to therapy withdrawal in up to 30% of patients [187,188,189]. In particular, digestive intolerance to thiopurines limits their use in 10–15% of patients [188,189]. However, it has been suggested that azathioprine and mercaptopurine could be interchangeable. Thus, an alternative strategy for managing azathioprine intolerance, mainly due to nausea or vomiting, is to switch to mercaptopurine (or vice versa).

Kennedy et al. performed a meta-analysis to determine the tolerance rate when prescribing mercaptopurine in azathioprine-intolerant patients and demonstrated that transitioning to mercaptopurine was a safe treatment strategy for more than two-thirds of patients who were intolerant to azathioprine [190]. This was particularly evident when the reason for azathioprine intolerance was gastrointestinal disturbance or hepatotoxicity, two of the most common causes for discontinuing thiopurine therapy [190]. However, switching from one thiopurine to another is usually not a good option in the case of flu-like illness, acute pancreatitis, or bone marrow aplasia [191,192].

### 7.3. Thiopurines Should Always Be Stopped and Non-Thiopurine Therapy Used Instead if Liver Abnormalities Are Detected

Abnormalities such as acute hepatocellular and cholestatic hepatitis have both been observed during thiopurine therapy [193]. A small percentage of patients may exhibit slight alterations in liver tests without clinical implications, which often return to normal parameters during follow-up, indicating that a dose adjustment of the immunomodulator is not always necessary [194,195].

When abnormalities in liver tests are more marked but not accompanied by jaundice, the dose of azathioprine or mercaptopurine may be reduced by 50%. It is usually unnecessary to completely withdraw these medications; however, strict and frequent clinical and analytical monitoring should be performed after reducing the dose. With this strategy, liver tests often normalize, thus allowing a cautious reintroduction of the initial dose of azathioprine or mercaptopurine [196,197,198].

If liver tests do not return to normal values with thiopurine tapering, it is recommended that therapy be withdrawn, which is necessary in less than 5% of patients. However, if azathioprine was initially prescribed, an alternative approach is to use mercaptopurine instead [190,199,200,201,202].

Nevertheless, it should be noted that in rare cases, thiopurines may induce severe cholestatic jaundice, which, unlike acute hepatocellular hepatitis, may not regress and can even progress despite thiopurine withdrawal [193]. Therefore, these drugs should be completely withdrawn, not merely tapered, in patients who present with clinically significant jaundice during thiopurine treatment [193].

### 7.4. Thiopurines Should Always Be Discontinued if Myelotoxicity Is Detected

It has been reported that mild leukopenia can resolve spontaneously without a change in dosage; therefore, in this case, the previous azathioprine/mercaptopurine dosage may be maintained with close monitoring [203]. The precise cut-off values for leukocyte or neutrophil counts that indicate when to lower the dose or discontinue the drug are still unknown. Some conservative authors suggest reducing the thiopurine dose (e.g., by 50%) when the leukocyte count is <4 × 10⁹/L, while others recommend this reduction when the count is <3 × 10⁹/L [203]. However, the risk of myelotoxicity is more closely related to neutropenia than to the total leukocyte count. Neutropenia is generally defined as an absolute neutrophil count of less than 1.5 × 10⁹/L [203]; thus, this figure appears to be a more appropriate cut-off for deciding on dose modification.

In cases of mild neutropenia, with an absolute neutrophil count between 1.0 and 1.5 × 10⁹/L, a dose reduction (e.g., to 50%) may be sufficient to resolve leukopenia, as demonstrated in some studies [203,204]. Nonetheless, some authors have observed that after reducing the thiopurine dose by 50%, it may be safely increased back to 100% once leukocyte values have normalized [205,206]. In cases of leukopenia relapse, the dose should be reduced permanently and individualized with great care. As the risk of infection increases significantly at an absolute neutrophil count below 1 × 10⁹/L [193], it is prudent to stop thiopurine administration (rather than just decrease the dose) in patients with lower counts [204].

### 7.5. Withdrawal of Thiopurines (When Administered as Monotherapy) Should Be Strongly Recommended in All Patients after Several Years in Remission

Thiopurine immunomodulators are an effective maintenance therapy for both CD and UC [111,116]. However, some studies have underscored the risks associated with the long-term use of these drugs [82,207,208]. Therefore, the periodic re-evaluation of the risk/benefit ratio of continued treatment with thiopurines is crucial. With the acknowledgment that IBD is a chronic condition requiring long-term therapy, it is increasingly recommended to continue effective maintenance therapy [209]. However, considering the risk of significant adverse effects, along with the necessity for long-term therapy in patients who are frequently young, the idea of discontinuing thiopurines in a patient in remission remains attractive [209].

A small retrospective study published in 1996 suggested that the withdrawal of azathioprine might be considered in patients who had maintained complete remission without steroids for longer than 3.5 years, as the 2-year relapse rate appeared to be similar whether the treatment was continued or stopped after this time [210]. However, subsequently, a multicenter, randomized, double-blind withdrawal trial was conducted, in which patients who were in clinical remission on azathioprine for more than 42 months were randomized to continue azathioprine or receive an equivalent placebo for 18 months [211]. The relapse rates at 18 months were 8% and 21%, respectively, indicating that azathioprine withdrawal was not equivalent to continued therapy for maintaining remission in patients with CD who had been in remission on azathioprine for over 3.5 years. Therefore, the authors concluded that azathioprine maintenance therapy should be continued beyond this time period [211]. In fact, at 5 years post-withdrawal, the cumulative relapse risk in the withdrawal group was as high as 63% [212].

Three subsequent randomized controlled trials also showed higher relapse rates in the drug withdrawal arm, which ranged from 17% to 53% at 12 months and were 31% at 24 months [213,214,215]. A subsequent meta-analysis of CD studies showed that continuing thiopurines reduced the relapse risk at 1 and 5 years with pooled odds ratios of 0.25 and 0.53, respectively [216].

Only one multicenter double-blind randomized controlled trial of azathioprine withdrawal in UC patients has been reported. The one-year relapse rates were 59% with azathioprine withdrawal and 36% with continued therapy (a statistically significant difference) [217]. In a multicenter retrospective study, one-third of UC patients relapsed within 12 months after azathioprine withdrawal, and two-thirds relapsed within 5 years [218]. Cohort studies reported varied relapse rates after immunomodulator withdrawal: from 11% to 77% at 12 months, from 43% to 65% at 5 years, and up to 87% with longer follow-up periods [194,219].

In conclusion, even after a long duration of clinical remission under thiopurines, the withdrawal of these drugs is associated with a high risk of relapse [220]. Therefore, thiopurine indefinite maintenance therapy should be at least considered in patients with IBD once remission has been achieved. When balancing the overall risks and benefits of prolonged maintenance therapy with thiopurines, it is likely that some clinicians and patients will accept the relatively small risk of lymphoid malignancy and opportunistic infections to prevent the ongoing morbidity and impact on quality of life associated with the chronic symptomatic activity of IBD.

## 8. Anti-TNF Agents

### 8.1. Anti-TNFs Are Not Useful for Treating Stricturing CD, Which Will Always Require Endoscopic Dilation or Surgery

CD typically causes inflammatory lesions in the ileocolonic region, but up to half of patients will develop complications such as strictures over time [221]. Thus, many patients experience disease progression leading to stricturing lesions, as no current drugs effectively prevent or reverse established fibrosis. Consequently, these patients are often treated with surgery or endoscopic balloon dilation [221].

Although fibrotic lesions are (almost) always associated with some degree of inflammation, there is limited evidence supporting the use of medical therapy in this context. Initially, some studies (a small cases series published only as an abstract) suggested that the healing process of inflammatory lesions might result in the formation of strictures [221]. However, a later analysis based on the TREAT registry found that the risk of stricturing complications was similar between patients treated with infliximab and those who were not [222]. In fact, over the past two decades, data from several cohorts have been published, indicating a clinical benefit associated with anti-TNF drugs [221].

A prospective, open-label observational study, known as the CREOLE study, focused on the efficacy of anti-TNF treatment specifically for patients with established symptomatic stenosis [223]. In this landmark research, 97 patients were evaluated over a 24-week period to assess the success rate of adalimumab treatment. Nearly two-thirds of the strictures were located in the ileum, with 13% of lesions situated at the ileocolonic anastomosis. At 24 weeks, 64% of the patients achieved treatment success, which was sustained in 29% of the patients during the long-term 4-year observation period [223].

In a more recent study, 262 patients with symptomatic stricturing CD who were receiving their first anti-TNF therapy (infliximab or adalimumab) and had no prior history of biological, endoscopic, or surgical therapy were included [224]. Anti-TNF treatment was effective in 87% and 73% of the patients after 6 and 12 months, respectively, and remained effective in 26% after a median follow-up of 40 months.

### 8.2. De-Escalation of Anti-TNF Treatment (Either Reducing the Dose or Increasing the Administration Interval) in IBD Is Generally Recommendable

Biologic therapy stands as an effective treatment for IBD; however, due to potential cost and safety concerns, de-escalation strategies, primarily for anti-TNF agents, have been proposed, especially following previous dose intensification. In clinical practice, approximately one-third of patients in remission after dose intensification revert to standard dosing. Conversely, de-escalation from standard dosing of anti-TNF agents is generally uncommon [225].

Around one-third of patients subjected to anti-TNF de-escalation, either from previous dose intensification or from standard dosing, experience relapse [225]. However, interpreting these relapse rates accurately is challenging due to the absence of a control group in most cases, although it seems that for some patients, the risk indeed increases [225]. Notably, the first (and only) randomized controlled trial comparing extended dosing intervals of adalimumab with standard intervals in stable CD patients (the LADI trial) reported similar persistent flare incidence in both groups, although the de-escalated group exhibited less clinical and biochemical remission and required more rescue therapy [226].

Predictive factors for relapse post-de-escalation remain unclear, making decision making challenging. However, the risk of relapse appears to be lower for patients in clinical, biologic, and endoscopic/radiological remission at de-escalation. Conversely, de-escalation should be approached cautiously or avoided altogether in high-risk patient groups, such as those with perianal fistulae or multiple prior surgeries [225]. Finally, it should be taken into account that, even though re-intensification in relapsed patients is usually effective, re-achieving remission is not guaranteed [225].

The main theoretical arguments favoring dose de-escalation are improved safety and cost savings, yet except for some cases, it has not consistently demonstrated a safer profile. Moreover, the cost-effectiveness of this strategy remains uncertain, with medication costs reduced but potential increases in non-medication healthcare costs. Additionally, the evolving landscape of biosimilars is altering the cost–benefit dynamic of de-escalation over time [225].

Prospective studies, preferably randomized controlled trials, with larger cohorts and longer follow-ups, are warranted to clarify the efficacy and safety of biologic de-escalation and identify optimal candidates for this strategy. Meanwhile, shared decision making with patients, weighing the pros and cons of de-escalation on a case-by-case basis, is paramount.

## 9. Extraintestinal Manifestations

### 9.1. In Hospitalized UC Patients, Thromboprophylaxis Is Not Indicated, as They Are Usually Young (and Therefore at Low Risk) and Have Rectal Bleeding (Which Could Worsen with Anticoagulation)

The extent and severity of intestinal involvement are related to the occurrence of thromboembolic complications, which often coincide with episodes of IBD activity [227,228]. Therefore, patients with IBD who are hospitalized for a flare are at a markedly increased risk of venous thromboembolism. This complication represents an important and preventable cause of morbidity and mortality in patients with IBD [229]. Therefore, systematic prophylaxis with low-molecular-weight heparin is recommended for all IBD patients admitted for a flare [184,230,231].

While UC typically manifests with rectal bleeding, initiating prophylactic treatment with low-molecular-weight heparin is also advisable in this case, even though this approach may seem counterintuitive. This concern leads to the lower utilization of pharmacological thromboprophylaxis, especially in patients with overt bleeding [232]. However, the use of prophylactic heparin has not been associated with an increased risk of major or minor bleeding or the need for blood transfusion in patients with IBD [233]. Nevertheless, it is evident that this medication should be used cautiously in patients with severe bleeding, as a case of massive bleeding in a patient with corticosteroid-refractory UC attributed to the administration of low-molecular-weight heparin has been reported [234].

### 9.2. Ocular Manifestations of IBD Are Never an Emergency, and Therefore, Patients Experiencing Them Should Be Referred to an Ophthalmologist for Deferred Outpatient Evaluation

Two fundamental types of ophthalmologic manifestations have been described in IBD [235]. The first is a “benign” involvement, which includes processes such as conjunctivitis, scleritis, or episcleritis. All of these present as the so-called “red eye” and clinically produce a sensation of a foreign body, but they are not accompanied by ocular pain or loss of vision [236]. These mild ocular manifestations usually respond favorably to the basic treatment of IBD and, if necessary, topical steroids can be administered [236]. Conversely, ophthalmologic manifestations can become severe, as is the case with uveitis, a complication described in 0.5–3% of patients with IBD [236]. Uveitis manifests as visual disturbances (blurry vision or decreased visual acuity), ocular pain, photophobia, and a headache [236]. Unlike conjunctivitis or episcleritis, the course of uveitis is usually independent of the activity of intestinal disease [236].

The early diagnosis and treatment of uveitis are essential, preventing complications such as irreversible vision loss, so this extraintestinal manifestation should be considered an ophthalmologic emergency. Uveitis can be difficult to differentiate from conjunctivitis or episcleritis by a nonspecialist physician. Therefore, a patient with IBD and ocular manifestations should be evaluated on an urgent basis by an ophthalmologist and should not be referred for deferred evaluation by this specialist [184].

## 10. Anemia

### 10.1. Anemia (i.e., Low Hemoglobin Levels), Not Iron Deficiency (i.e., Low Ferritin Levels), Is the Only Significant Laboratory Finding

The prevalence of anemia in patients with IBD is very high, although the reported figures vary significantly between 10% and 75% [237,238]. There are several types of mechanisms involved in the development of anemia in patients with IBD, the most common being secondary iron deficiency due to continuous blood losses in the gastrointestinal tract [239].

Anemia is just one aspect of the condition, as iron deficiency can cause symptoms even when fully developed anemia is not yet present [237]. Iron deficiency, with or without anemia, is a relevant analytical parameter in IBD. In fact, it is quite common in everyday clinical practice to find iron deficiency as the only sign of disease activity in IBD patients [239].

The decision to supplement iron in patients with iron deficiency but without anemia is not entirely clear and may depend on the clinical scenario and individual preference. The arguments for treating isolated iron deficiency are based on the fact that iron is essential for all cells of the body, and symptoms of iron deficiency are not only anemia-specific (such as fatigue and shortness of breath). Iron deficiency also affects nail growth, skin health, and mucosal regeneration and may cause symptoms such as headaches, sleep disorders, decreased libido, erectile dysfunction, and many more, including deterioration in quality of life [240,241]. Furthermore, recent evidence suggests that body iron levels should also be within the normal range, after iron supplementation, to fully improve cognitive performance and quality of life [242,243,244]. Finally, it is important to note that untreated iron deficiency is likely to progress to iron deficiency anemia [237].

### 10.2. The Impact of Anemia on the Quality of Life of Patients with IBD Is Quite Limited

The impact of iron deficiency anemia on quality of life is often underestimated or even ignored. However, the truth is that the repercussion of anemia on the quality of life of both general patients [245,246] and, specifically, patients with IBD [247,248,249,250,251] is substantial. In fact, the impact of anemia on the quality of life of these patients can be similar to that of a cancerous disease [247]. In addition, chronic fatigue resulting from anemia can weaken, affect, and worry these patients as much as abdominal pain or diarrhea [248]; therefore, the beneficial impact on quality of life derived from correcting anemia in patients with IBD can be similar to that of controlling diarrhea [248,250,252]. Moreover, anemia may impair quality of life even in the absence of specific symptoms [250,253]. For a long time, it was thought that the clinical symptoms of anemia (such as fatigue, headache, dizziness, shortness of breath, or tachycardia) occurred only when hemoglobin levels dropped abruptly [247,248]. It had been argued that patients would adapt to low hemoglobin levels if anemia developed slowly. This has led to the concept of “asymptomatic” anemia. In truth, the term “asymptomatic” seems to reflect the fact that impairments in physical condition, quality of life, and cognitive function may be unrecognized by both patients and their doctors. Therefore, the process of adaptation to chronic anemia would be, in fact, an adaptation to a lower quality of life [247,248].

### 10.3. Since Mild Anemia Is Common in Patients with IBD and Its Clinical Impact Is Only Evident When the Anemia Is Severe, Iron Therapy Is Rarely Necessary

The high frequency of low, albeit not excessively low, hemoglobin levels in patients with IBD often leads to an underestimation of this analytical alteration by physicians. One should not make the mistake of assuming that a certain level of anemia is a normal finding in patients with IBD and therefore does not require treatment [237,247]. On the contrary, oral iron administration should begin as soon as anemia is detected, defined according to the World Health Organization (WHO) as hemoglobin < 13 g/dL in males and <12 g/dL in females. Similarly, the therapeutic goal of oral iron therapy should be to completely correct the anemia, not just partially raise hemoglobin levels [184,239]. In fact, it is important to remember that the most significant improvement in quality of life is observed, precisely, when hemoglobin levels rise from 11 to 13 g/dL [254].

### 10.4. When Administering Oral Iron Treatment, Higher-Than-Usual Doses Should Be Used Because Its Absorption Is Often Decreased in Patients with IBD

Although it has been suggested that up to 200 mg of elemental iron per day are necessary to correct iron deficiency anemia, this is likely incorrect [255]. Since only approximately 10 mg of oral iron can be absorbed daily, higher doses are questionable. There is no rationale for using high doses of iron to treat iron deficiency anemia, whether in IBD or other associated diseases [256]. In fact, controlled efficacy studies on oral iron treatment in iron deficiency anemia among adults, the elderly, pediatric patients, and pregnant women, support the use of low-dose oral iron supplements [255,256,257,258]. From a physiological perspective, the iron absorption process is highly efficient but saturable [255,259]. A single tablet of most ferrous salt preparations (e.g., sulfate) provides more iron than the intestine can absorb in one day [256,257]. Non-absorbed iron salts can be toxic to the intestinal mucosa and may potentially activate the disease [237,260,261]. Furthermore, high doses of iron can cause diarrhea, impairing quality of life and complicating differentiation from an IBD relapse [260,262]. Therefore, as the absorption and efficacy of oral iron do not increase with higher doses, oral iron should be recommended at low doses (e.g., 50–100 mg of elemental iron daily), and higher doses would only increase the risk of adverse gastrointestinal effects (e.g., nausea, vomiting, constipation, and diarrhea) [239].

### 10.5. In Patients with IBD, Intravenous Iron Administration Should Be Reserved for Cases of Severe Anemia (e.g., Hemoglobin < 8 g/dL)

Following a widely agreed-upon algorithm, the initial therapeutic strategy for iron deficiency anemia in IBD patients is based on hemoglobin levels. Patients with hemoglobin levels above 10 g/dL could start treatment with oral iron. Those with levels below 10 g/dL—generally considered as severe anemia—should receive intravenous iron as the treatment of choice [237,239,263]. Intravenous iron should also be prescribed to patients with hemoglobin levels above 10 g/dL if there is intolerance to oral iron, failure to respond to oral iron treatment, or clinically active IBD [239].

## 11. Elderly Patients

*In Elderly Patients with IBD, the Use of Biological Drugs Should Be Avoided at All Costs*

While IBD typically affects younger individuals, elderly patients are increasingly represented in the IBD population [264]. Managing IBD in older patients can pose challenges due to their potential increased vulnerability to adverse events [264,265]. Partly due to the perception of less severe disease and concerns about therapy-related complications, effective immunosuppressive treatments are—erroneously—utilized less often in older patients compared to their younger counterparts. Multiple prior studies have noted that older patients with IBD are less likely to be prescribed anti-TNF therapy [266,267,268,269,270]. This results in prolonged morbidity from active disease in the elderly [266,267], while disease activity itself might increase the likelihood of more adverse events in this population [271].

Furthermore, multiple studies have noted that older patients with IBD are more likely to discontinue anti-TNF therapy [266,267,272,273,274,275,276]. The main reasons for stopping treatment reported in these cohorts included a lack of response and adverse events [275]. Earlier smaller studies have shown a higher risk of infections, hospitalizations, neoplasms, and/or mortality with anti-TNF therapy among elderly patients compared to younger ones [277]. However, the majority of available data come from retrospective observational studies, which may be constrained by confounding bias. Cheng et al. conducted a pooled analysis of data from randomized trials to assess the impact of age on the safety of anti-TNF therapy, showing that treatment of older patients with these agents did not increase the risk of serious adverse events or infections, compared with younger patients [278]. Although older anti-TNF users had numerically higher rates of infections than younger patients, this difference was not specific to biologics, and a similar numeric difference was also noted among those on the placebo [278]. Furthermore, anti-TNF therapy was similarly effective in older and younger patients [278]. Finally, elderly patients with IBD have a similar risk of developing infliximab-related immune-mediated adverse events and a loss of response compared with younger patients [279].

On the other hand, when deciding to start anti-TNF therapy in older individuals, clinicians should also consider the implications of untreated disease and the potential risks linked to alternative treatments like surgery, along with the probability of post-operative complications [264]. Furthermore, it is important to consider the risks of disease relapse from discontinuing biological treatment and that the acute use of corticosteroids in elderly patients is also associated with a higher risk of adverse events. Therefore, treatment strategies for older patients that minimize steroid exposure should be considered [280].

In summary, the principles of medical management of IBD in elderly patients should be generally the same as in other age groups [275] Thus, elderly IBD patients—a clinical distinction must be made between fit elderly patients and frail elderly patients—should be candidates for treatment with all of the therapeutic options available for younger IBD patients [264].

## 12. Pregnancy

### 12.1. During Pregnancy, Endoscopic Examinations Should Not Be Performed Even if They Are Clearly Indicated, Due to the Risk of Harming the Fetus

Theoretically, the insertion of a colonoscope in a pregnant woman could induce premature labor. However, although experience evaluating the safety of endoscopic examinations during pregnancy is very limited, it appears that this technique is well tolerated by both the mother and the fetus [281]. Specifically, flexible sigmoidoscopy does not induce labor or cause fetal malformations, so this technique is not contraindicated during pregnancy [281,282,283]. Furthermore, this safety seems to be independent of the gestational age [283,284]. In addition, a systematic review determined that a lower endoscopy carries a minimal risk for both the mother and child throughout any of the three trimesters of pregnancy [285].

Obviously, for any patient but especially in pregnant women, diagnostic tests should not be performed if their result will not change the therapeutic approach. However, sometimes the information derived from a rectosigmoidoscopy is useful for establishing the cause of symptoms and the corresponding treatment, as in pregnant women presenting with hematochezia or unexplained diarrhea, who, thanks to this technique, can be finally diagnosed with IBD. In this regard, a study involving a sigmoidoscopy during pregnancy demonstrated that the most frequent diagnosis was IBD and that this knowledge led to a substantial change in the treatment of these women [286]. Similarly, a rectosigmoidoscopy would be indicated in patients with a known diagnosis of IBD who do not improve with standard treatment. Accordingly, the ECCO guidelines on pregnancy state that “during pregnancy, endoscopy can be performed when needed to guide clinical decision making” [287].

### 12.2. In Pregnant Women, Due to the Risk that Medications Pose to Fetuses, Efforts Should Be Made to Administer the Minimum Possible Treatment for IBD, Even if It Means that Some Intestinal Activity Persists

Since IBD typically affects young patients during their reproductive years, issues related to pregnancy often arise for them. The use of medications during pregnancy is a common concern for both the patient and the treating physician.

In a study examining the views and perceptions of women with IBD regarding medication use during pregnancy, a substantial portion (36%) of the participants believed that all IBD medications could negatively impact the health of unborn children [288]. Contrary to established evidence, around 24% of participants endured their symptoms without taking medication, because of the misconception that IBD medications are harmful [288]. Another study highlighted the concerns of women with IBD regarding fertility, pregnancy management, and the postpartum period, even though they received regular obstetric and specialized IBD care [289]. The most significant worry among participants was the potential impact of their medication on pregnancy and offspring. They specifically feared the effects of medication on the child’s immune system [289].

On the contrary, since it is clearly demonstrated that IBD activity during pregnancy is associated with a higher risk for the newborn (such as a premature birth or a low birth weight), it is important to “aggressively” treat IBD flare-ups during pregnancy instead of adopting a falsely “conservative” approach by arguing that the medications used in treating this disease may be harmful to the fetus [290,291,292,293,294]. In summary, the best way to ensure the fetus’s well-being is to effectively control the mother’s IBD activity [287].

Pregnant women experiencing a flare should be managed according to current guidelines for non-pregnant patients, with 5-ASA, steroids, anti-TNF agents, ustekinumab, or vedolizumab. Initiating monotherapy with a thiopurine is generally not recommended due to the slow onset of action and the potential risk of adverse events. Current guidelines recommend avoiding JAK inhibitors and S1P receptor modulators during pregnancy [287].

### 12.3. Biological Agents Are Not Safe during Pregnancy, and Therefore, They Should Be Discontinued before the Third Trimester

Biologics for the treatment of IBD are immunoglobulin G1 (IgG1) full monoclonal antibodies. In early pregnancy, only insignificant amounts of IgG are transported by passive diffusion. However, the maternal transfer of IgG1 through placental Fc neonatal receptors starts at weeks 13–17 and significantly increases thereafter. This transfer can result in cord blood levels in infants that may be several times higher than those in maternal serum [295]. Furthermore, detectable biological agents may persist in the infant’s blood for up to 12 months [296].

Acknowledging the active transfer of biologics and the potential exposure of infants in utero and in early life (a sensitive period for immune system programming and development), there is a theoretical concern that such exposure may disturb the child’s immunity. Discontinuing a biologic drug before the third trimester would limit the fetus’s exposure to the drug and therefore could be beneficial to reduce its detrimental effects. However, a follow-up of children exposed to anti-TNF agents in utero showed no differences in infection rates requiring hospital admission, milestone developments, or other negative outcomes between those exposed only during early trimesters and those exposed throughout all three trimesters [287,290,291,292,293,294].

Furthermore, stopping a biologic drug that has induced remission may increase the chances of relapse, with negative consequences for both the mother and fetus [297,298,299]. Additionally, a prolonged drug (anti-TNF) holiday may increase the likelihood of a secondary loss of response in the postpartum period [287]. Therefore, in clinical practice, for women in remission, it is advised to continue anti-TNF agents during pregnancy since the potential risks of active disease are likely greater than those associated with anti-TNF use [287]. However, if a pregnant patient in long-term remission wishes to discontinue anti-TNF prior to the third trimester, the resumption of anti-TNF use shortly after delivery is recommended [287].

Other biological agents besides anti-TNFs, such as vedolizumab and ustekinumab, are also IgG1 [292,293]. Consequently, when the mother is treated during pregnancy, the fetus is exposed to these drugs from the second trimester, because from this time onwards, they cross the placenta. The clearance time of these drugs in newborns varies, but generally, vedolizumab and ustekinumab are cleared faster than anti-TNFs [292,293]. Animal studies have not shown a risk of teratogenicity with these drugs, and data are increasingly suggesting that both vedolizumab and ustekinumab are safe during pregnancy in humans as well [292,293,294].

### 12.4. Breastfeeding Is Contraindicated While the Mother Is Undergoing Treatment with Biological Agents

Breastfeeding is generally considered low-risk for patients on currently approved biologic drugs (mainly anti-TNFs, vedolizumab, and ustekinumab), as IgA is the predominant immunoglobulin found in breast milk, while the biologic agents used to treat IBD are IgG. Therefore, secretion and transfer in breast milk should be minimal [292]. Furthermore, due to degradation in the infant’s digestive tract, exposure to these drugs is unlikely to have any clinical relevance [293]. In summary, drugs that are considered low-risk during pregnancy are also considered low-risk during breastfeeding and thus can be continued [287]. In particular, several studies have shown that breastfeeding during treatment with anti-TNF agents appears to be safe and should not be discouraged [287,293]. Regarding non-anti-TNF biologics, the most relevant data on their safety during breastfeeding are summarized below.

In a study on monkeys, vedolizumab was detected at low concentrations in the breast milk of 3 out of 11 animals 28 days after their birth [300]. The first data on humans were reported in a study involving five breastfeeding women with IBD [301]. Serum and breast milk samples were collected before infusion, 30 min later, and over the following 14 days. The lowest vedolizumab concentrations (ranging from 0.124 to 0.228 mg/mL) were detected in breast milk samples collected before the infusion, peaking at 0.318 mg/mL on days 3 through 7, a concentration estimated to be less than 1% of serum levels. Considering the amount of milk ingested by a baby, the maximum amount of vedolizumab received is estimated to be 0.048 mg/kg per day [301]. Another recent study found similar findings in five post-partum women on maintenance therapy with vedolizumab [302]. Serum and breast milk samples were collected after 1 h and on the following days after the infusion. The amount of vedolizumab detected in breast milk was about 1% of the corresponding serum sample, peaking 3–4 days after the infusion and then progressively declining [302]. More recent data from 11 nursing women showed an average milk concentration of approximately 0.13 µg/mL with a peak of up to 0.56 µg/mL 3–4 days after the infusion [303].

Studies in macaques have shown that ustekinumab concentrations in breast milk are about 1/1000 of the serum blood concentration, a level considered too low to result in the systemic immunosuppression of the child [293]. Matro et al. reported on the concentration of various biologics in breast milk from patients included in the PIANO registry [304]. In a cohort of 824 infants, breastfeeding while receiving biological therapy did not affect the rate of infection or developmental milestones compared with non-breastfeeding. Six patients treated with ustekinumab provided breast milk samples, and ustekinumab was detected in four (67%) samples, with peak concentrations between 12 and 72 h after injection (range: 0.72–1.57 µg/mL). The authors concluded that lactation is compatible with maternal biologic therapy, including ustekinumab, based on minimal transfer rates in breast milk and no association with infant infections or developmental milestones [304]. Finally, Saito et al. also analyzed ustekinumab concentrations in breast milk, finding levels at 1/1400 of maternal serum, similar to previous studies on macaques and other case studies with CD [305].

### 12.5. In Children Exposed In Utero to Biologics, Non-Live Inactivated Vaccines Are Less Effective and Safe

Some reports and a meta-analysis suggest that several non-live inactivated vaccines (hepatitis A, hepatitis B, influenza, and *Streptococcus pneumoniae*) may not elicit adequate seroprotection when administered to adult IBD patients treated with anti-TNF agents [83,306,307,308,309]. In contrast, vaccines administered to children with IBD generally achieve adequate immunogenicity, regardless of the treatment, including anti-TNF agents [310,311].

Studies evaluating the efficacy of inactivated vaccines given to infants exposed to biologics (mainly anti-TNF agents) in utero suggest that the response (seroprotection) to inactivated vaccines could be considered adequate in most infants, although the available information is limited [312]. A systematic review and meta-analysis of studies assessing pregnancy and neonatal outcomes of women with immune-mediated inflammatory diseases (including IBD, rheumatoid arthritis, and psoriasis) exposed to anti-TNF agents during pregnancy demonstrated an adequate immune response to tetanus, *Streptococcus pneumoniae*, diphtheria, and hepatitis B virus [313]. In summary, newborns with a history of in utero exposure to anti-TNF agents should adhere to a standard vaccination schedule for inactivated vaccines, as their effectiveness appears to be adequate based on current evidence [312].

Regarding the safety of inactivated vaccines, a systematic review has confirmed their safety in children with chronic conditions treated with biologics [314]. Furthermore, immunizations against hepatitis B and pneumococcus are well tolerated both in children and adults with IBD who are prescribed anti-TNF therapy [310,315,316,317]. When the studies evaluating the safety of non-live inactivated vaccines administered to infants exposed to biologics in utero were reviewed [312], no or only minor adverse events were reported following vaccination against hepatitis B virus, *Haemophilus influenzae*, influenza, and diphtheria [318,319]. Similarly, a meta-analysis of studies involving children born to mothers with immune-mediated inflammatory diseases exposed to anti-TNF agents during pregnancy reported only minor adverse events related to vaccinations, including tetanus, *Streptococcus pneumoniae*, diphtheria, hepatitis B virus, and *Haemophilus influenzae* type B [313]. In summary, there is no recommendation to alter the vaccination schedule for inactivated vaccines in infants exposed to biologics in utero, as this population does not appear to experience significant adverse events related to these vaccinations [287,320].

### 12.6. In Children Exposed In Utero to Biologics, All Live-Attenuated Vaccines Are Safe

Due to the risk of disease from uncontrolled replication, severe immunosuppression is generally considered a contraindication for live-attenuated vaccines [312,321]. Live-attenuated vaccines commonly administered in clinical practice during the first 12 months of life (when serum levels of biological drugs in the child can be detected) include the rotavirus vaccine and, in some countries, the Bacillus Calmette–Guérin (BCG) vaccine. Since the trivalent MMR (measles, mumps, and rubella) vaccine dose is recommended between 12 and 15 months, there is generally no reason to delay vaccination [40]. Studies assessing the safety of live-attenuated vaccines administered to infants exposed to biologics in utero have generally reported no serious adverse events [312]. However, despite the lack of severe complications following rotavirus vaccination in this context, five fatal cases of disseminated BCG infection in infants exposed to anti-TNF agents in utero, including infliximab and adalimumab, have been reported [312].

In accordance with ECCO recommendations, in children exposed in utero to biologics, vaccines should be withheld within the first year of life or until the biologic is no longer detectable in the infant’s blood [287]. While avoiding the BCG vaccine may not be critical in most developed countries, it poses a more difficult decision in countries with a high tuberculosis incidence, where determining the serum levels of biological drugs in the child may be challenging or impossible. Therefore, the decision should always be individualized and made on a case-by-case basis [312].

Regarding rotavirus vaccination, most recent studies suggest that the risk of vaccination in infants exposed to biological agents in utero appears to be minimal or nil [313,322,323]. Therefore, it is increasingly common to allow such vaccination if it is deemed necessary, especially in developing countries, where rotavirus-related mortality is significant. Nevertheless, the risk–benefit ratio must always be carefully considered [312].

### 12.7. Administration of a Live-Attenuated Vaccine to a Breastfed Infant While the Mother Is Receiving Anti-TNF Agents Is Not Recommended Unless Infant Anti-TNF Serum Levels Are Undetectable

In March 2022, the European Medicines Agency (EMA) issued a direct healthcare professional communication (DHPC) concerning the use of live vaccines in infants exposed to infliximab during breastfeeding. According to this DHPC, the marketing authorization holders of infliximab, in agreement with the EMA, conveyed controversial information. They stated that infliximab has been detected in breast milk at low levels and also in infant serum after exposure via breastfeeding. Consequently, the DHPC advised against administering live vaccines to breastfed infants unless the infant’s serum levels of infliximab are undetectable [312]. This recommendation aroused significant concern among gastroenterologists specialized in IBD, pregnancy, and lactation, leading to several responses opposing it [312,324,325,326]. Key arguments against the EMA recommendation included the following [312]:

(a) Multiple studies have consistently demonstrated that peak levels of infliximab in breast milk are minimal, typically less than 1% of maternal serum levels (see corresponding section above);
(b) A fully breastfed infant is estimated to receive a maximum of 0.045 mg of infliximab per kilogram of bodyweight per day [327]. Notably, breastfeeding while the mother is receiving infliximab treatment did not affect the clearance of infliximab in infants exposed in utero to the drug [312];
(c) The EMA’s recommendation was primarily based on a case report involving two mothers receiving infliximab while breastfeeding. One infant’s infliximab serum levels were undetectable, whereas the second infant’s levels were measured at 1.7 μg/L during maternal induction treatment, equivalent to approximately 2% of the maternal serum infliximab level at that time [328];
(d) The largest study on biological treatment during breastfeeding involved 29 women treated with infliximab. This study confirmed very low levels of infliximab in breast milk and demonstrated that breastfed infants of mothers using biologics, including infliximab, had similar risks of infection and rates of milestone achievement compared to non-breastfed infants or infants not exposed to biologics [304];
(e) In the most recent study evaluating the risk of serious adverse events associated with live-attenuated vaccines in children breastfed by mothers receiving biological therapies—the DUMBO registry [329]—a quarter of breastfeeding mothers were on biologics (mostly anti-TNF agents). Sixty-eight percent of these children breastfed for at least 6 months received the rotavirus vaccine, 97% received the first dose of the trivalent MMR (measles, mumps, rubella) vaccine if they were breastfed for at least 12 months, and 84% received the first dose of the varicella vaccine if they were breastfed for at least 15 months. No serious adverse events related to these live-attenuated vaccines were reported [330];
(f) The recommendation against administering live-attenuated vaccines during lactation if mothers are treated with infliximab can have significant adverse consequences [324,325,326]. Breastfeeding women may choose to forgo medical treatment, decide not to breastfeed, or delay infant immunization. Such decisions could result in missed or delayed crucial vaccinations during the early years of a child’s life, potentially increasing the risk of serious infections [312].

In conclusion, based on the available literature regarding the safety of live vaccines in infants breastfed by women receiving anti-TNF therapies, the benefits of breastfeeding while on infliximab (or any other anti-TNF agent) and adhering to national infant immunization programs likely outweigh any hypothetical risks to the infant [312].

## 13. Surgery

### 13.1. In CD, Surgery Always Represents the Failure of Medicine and Is Only Indicated When Medical Treatments Fail

Advances in medical management, combined with concerns shared by patients and doctors about the irreversibility of bowel resection, may lead some to view surgery as a last resort, to be delayed or avoided whenever possible, except in well-recognized situations of multiple medical treatment failure [331].

However, the LIR!C randomized controlled trial suggested that laparoscopic ileocecal resection was a viable and reasonable alternative to infliximab for patients with limited (diseased terminal ileum < 40 cm), non-stricturing, ileocecal CD who do not respond to conventional therapy [332]. This study indicated that while laparoscopic ileocecal resection was not superior to infliximab treatment, it was comparable in terms of restoring quality of life and was not associated with more serious adverse events. A long-term follow-up revealed that over one-third of patients initially treated with infliximab required an ileocecal resection within a few years, while only one in four patients who initially underwent resection needed subsequent anti-TNF therapy [333]. Furthermore, laparoscopic ileocecal resection was a cost-effective treatment option compared with infliximab [334]. Based on these findings, laparoscopic ileocecal resection should be offered as an alternative to anti-TNF therapy for patients with limited, non-stricturing ileocecal CD that does not respond to conventional therapy [332].

More recently, Agrawal et al. conducted a population cohort study to compare long-term outcomes in patients diagnosed with ileal and ileocecal CD who underwent either ileocolic resection or received anti-TNF therapy within one year from diagnosis [335]. They found that ileocolic resection was associated with a 33% reduced risk of systemic corticosteroid exposure and CD-related surgery compared to medical therapy. Additionally, 50% of patients who underwent ileocolic resection did not require any further medical therapy at 5 years [335].

### 13.2. In Patients with Acute Severe UC, Surgery Should Be Delayed as Much as Possible

Acute severe UC potentially carries a high risk of death [231]. However, the introduction of intravenous steroid treatment in 1955 reduced acute mortality from 24% to 7% [336]. Timely surgery combined with intensive medical therapy further decreased the mortality rate to less than 1% in specialized centers [337]. However, with the advent of rescue medical therapy for steroid failure—using cyclosporine or infliximab—the necessity of surgery is being re-evaluated [27].

At present, in patients with acute severe UC, surgery should be performed when clearly indicated (such as in cases of suspected perforation, a toxic megacolon, or refractory bleeding), when medical rescue therapy is contraindicated, or in cases of failure of medical rescue therapy [27]. However, surgery should be considered relatively early in the treatment process as a beneficial alternative, not just a fallback after unsuccessful medical management. Postponing surgery is linked to a higher risk of postoperative complications, making the timing of the surgery crucial. Therefore, surgery should preferably be performed in a semi-elective setting rather than an emergency one whenever possible, as the mortality rate following a colectomy for UC is higher in emergency situations [27].

Randall et al. evaluated the long-term outcomes following urgent colectomies for acute severe UC and investigated whether the duration of in-hospital medical therapy was related to postoperative outcomes [338]. Patients with a major complication at any time during their follow-up had a significantly longer duration of medical therapy before a colectomy was performed than patients with no major complications. Therefore, it was concluded that delayed surgery for acute severe UC was associated with an increased risk of postoperative complications. This result does not question the value of medical therapy, which should be pursued vigorously to avoid surgery [171]. However, if medical therapy is continued for too long, the complication rate increases if surgery becomes necessary. Therefore, the challenge for both physicians and surgeons is to monitor patients closely and make the decision to operate at an appropriate time. We should never forget that our primary aim should be to reduce patient mortality rather than save the colon [171].

### 13.3. Most Drugs Used in IBD Treatment (Corticosteroids, Thiopurines, Biologics, and Small Molecules) Equally Increase the Risk of Postoperative Complications

Treatment with corticosteroids represents a risk factor for the development of complications during and after surgery [339,340,341]. However, unlike steroids, treatment with thiopurines (or methotrexate) does not increase the risk of postoperative complications (infectious or otherwise) in patients undergoing surgery for IBD [342,343]. Currently, a significant number of patients undergoing surgery are receiving biological agents. Therefore, it is crucial to ascertain whether this treatment increases the risk of complications to make informed decisions regarding the scheduling of surgeries.

Initial findings from several meta-analyses suggested an increased risk of post-operative complications in IBD patients undergoing anti-TNF therapy, particularly in those with CD [344,345]. However, in contrast with these findings, recent meta-analyses focusing on CD or UC suggest that the preoperative administration of biological agents is not linked to increased early postoperative complications [346,347,348,349,350]. Additionally, prospective studies assessing this effect found no association between preoperative anti-TNF administration or drug levels and postoperative complications in IBD patients. Finally, the largest cohort study that has evaluated the safety of preoperative anti-TNF, vedolizumab, or ustekinumab treatments in IBD patients concluded that none of these drugs increased the risk of postoperative complications [351]. Accordingly, current guidelines suggest that preoperative treatment with any biological therapy, including vedolizumab and ustekinumab, does not increase the risk of post-operative complications in patients with IBD undergoing abdominal surgery [352]. Hence, the withdrawal of biological therapy before surgery may not be necessary (i.e., it is not mandatory) to reduce the incidence of postoperative complications [353]. It is likely that this same recommendation can be applicable to small molecules [350].

### 13.4. Previous Failure with an Anti-TNF Agent Necessarily Warrants Switching to a Drug with a Different Mechanism of Action (Such as Vedolizumab or Ustekinumab) to Prevent Post-Operative Recurrence of CD after Surgery

Anti-TNF therapy is frequently used in the treatment of refractory CD. Unfortunately, primary or secondary treatment failure of anti-TNF treatment is not uncommon [354,355]. Therefore, in clinical practice, a substantial proportion of patients who receive anti-TNF agents after surgery—to prevent post-operative recurrence (POR)—have been exposed to these agents prior to surgery. As the number of patients who do not respond to multiple biologics/small molecules and require surgery increases, the decision regarding postoperative treatment will become more complex [356,357].

Some studies have reported that anti-TNF agents are less effective for the prevention of POR in patients with previous exposure to anti-TNFs, compared with those naïve to these agents [358,359,360,361,362], suggesting that a reasonable approach to prevent POR would be choosing a biologic with an alternative mechanism of action (non-TNF-related) for those who experienced treatment failure with an anti-TNF agent [356]. Noteworthy, the aforementioned studies have some relevant limitations (including their retrospective design). However, other investigators have reached opposite conclusions, that is, that anti-TNF remains an effective option to prevent POR for patients operated upon with previous anti-TNF failure [363,364,365,366,367,368].

As the presence of intestinal complications is known to be one of the risk factors for the lower efficacy of anti-TNF agents, the requirement for surgery early after the initiation of anti-TNF treatment may not indicate the primary ineffectiveness of the agent but insufficient effectiveness owing to the presence of intestinal complications, thereby explaining the favorable results in the post-operative scenario despite previous anti-TNF failure [369]. Therefore, in these patients, the removal of intestinal complications by surgery might “reconstitute” the efficacy of anti-TNF agents. Others have tried to explain this by arguing that anti-TNF treatment shortly after surgery, when there are still no signs of active disease, could interfere with the initial pathogenic mechanisms of tissue damage, changing the natural evolution of the disease. Based on these results, some authors have proposed maintaining the anti-TNF treatment if these agents were used preoperatively, and then performing early screening to evaluate and adjust medications. This strategy might spare further possible biological treatment options in the future.

Nevertheless, the main limitation of all previously mentioned studies is that a control group, treated with a different biological agent than an anti-TNF, was not included. A preoperative anti-TNF therapy requirement might simply be a surrogate marker of a more severe, refractory disease (to any treatment) before surgery, and therefore, it may not necessarily imply a worse response when readministering anti-TNF treatment (compared with non-anti-TNF biological agents). In fact, both vedolizumab and ustekinumab are also less effective in anti-TNF-exposed patients [355]. Unfortunately, the two main strategies used to treat a patient with primary non-response to an anti-TNF agent—switching to a second anti-TNF or switching to vedolizumab/ustekinumab—have not been properly compared in randomized controlled trials [355].

Recently, some studies have compared, in a non-randomized manner, the efficacy of anti-TNFs with that of other biologics in preventing POR. Yanai et al. reported that the continuation of anti-TNF treatment after surgery resulted in a similar rate of endoscopic POR as switching to a different mechanism of action [370]. On the other hand, Le Cosquer et al. evaluated CD patients who underwent bowel resection after failure of at least one anti-TNF treatment [371]. The rates of POR at two years were lower (24%) in the patients treated with anti-TNFs than in those receiving other biologics such as ustekinumab or vedolizumab (45%).

## 14. Conclusions

Two facts seem clear on the subject of errors: they are very common in medical practice—and in particular, in the healthcare of IBD patients—and most of them can be prevented. Despite the existence of guidelines for both disease management and preventive aspects of IBD care, a considerable variation in clinical practice and a lack of adherence to clinical guidelines for IBD still remain. In the present review, we have identified some mistakes frequently observed in clinical practice in the management of patients with IBD, then we have reviewed the scientific evidence available on the subject, and finally we have proposed the most appropriate recommendations. There is a clear need for a greater dissemination of clinical practice guidelines among gastroenterologists and for the implementation of ongoing training activities supported by scientific societies. Finally, it is recommended that IBD patients be followed in specialized units, which will be associated with higher-quality healthcare and lower likelihood of errors in managing these patients.

## Figures and Tables

**Table 1:** Summary of the most common mistakes in managing patients with inflammatory bowel disease.

**DIAGNOSIS AND DIFFERENTIAL DIAGNOSIS**
✓ “When admitting previously diagnosed UC patients with rectal bleeding, it is not necessary to rule out an enteric infection as it is evident that it is a flare-up of their IBD”
✓ “Clostridioides difficile infection should only be considered in IBD patients who have recently received antibiotics”
✓ “Assume that all cases of proctitis are ulcerative proctitis”
✓ “The endoscopic lesions of UC are always continuous”
✓ “In severe UC flare-ups, a complete colonoscopy is necessary to precisely define the extent of the disease and choose the most appropriate treatment”
✓ “An obstructive picture in patients with CD is always due to intestinal stenosis as a consequence of their underlying disease”
✓ “The clinical manifestations of toxic megacolon are very characteristic, so its diagnosis is usually straightforward”
✓ “CMV infection, whenever present, always plays a causative role in the flare-up of UC or in the episode of corticosteroid refractoriness”
**PREVENTION**
✓ “For patients with CD who smoke, repeatedly emphasizing the necessity of quitting smoking may not be so crucial”
✓ “Early screening for latent tuberculosis is not necessary, it is sufficient to screen only when the patient already requires immunosuppressive treatment”
✓ “Routinely assessing the need for vaccination at the time of diagnosis is not necessary in patients with IBD”
**NUTRITION AND DIET**
✓ “Self-imposed food restrictions help prevent the onset of IBD flare-ups and aid in controlling their activity”
✓ “Patients admitted for an IBD flare benefit from complete fasting, as it reduces disease activity. The administration route for nutritional supplements should be parenteral, as it is more effective and better tolerated than enteral feeding”
**5-AMINOSALICYLATES**
✓ “Aminosalicylates are equally effective for treating CD and UC”
✓ “The combination of oral and topical aminosalicylates is deemed unnecessary, as each treatment alone demonstrates similar efficacy”
✓ “The total dose of aminosalicylates should be split into at least two daily administrations, as a single daily dose is less effective”
**CORTICOSTEROIDS**
✓ “Corticosteroids are generally used appropriately (only when necessary)”
✓ “Corticosteroids are effective in patients who are already receiving treatment with immunomodulators or biological agents”
✓ “It is recommended to start with low or intermediate doses of corticosteroids, and only use full doses if no response is observed”
✓ “At least 10 days must be waited before considering a patient with severe UC treated with intravenous corticosteroids as corticosteroid-refractory”
✓ “Faced with a patient with severe UC resistant to corticosteroids in whom a CMV infection is detected and antiviral treatment is initiated, it is necessary to immediately and completely discontinue the steroids”
✓ “Since bone loss does not begin to occur until several months after the start of corticosteroid treatment, it is not necessary to initially administer prophylactic therapy for osteopenia”
**THIOPURINES**
✓ “It is advisable to split the dose of thiopurines into several intakes to facilitate gastric tolerance”
✓ “In patients who develop digestive intolerance to azathioprine, thiopurine drugs should be permanently discontinued”
✓ “Thiopurines should always be stopped and non-thiopurine therapy used instead if liver abnormalities are detected”
✓ “Thiopurines should always be discontinued if myelotoxicity is detected”
✓ “Withdrawal of thiopurines (when administered as monotherapy) should be strongly recommended in all patients after several years in remission”
**ANTI-TNF AGENTS**
✓ “Anti-TNFs are not useful to treat stricturing CD, which will always require endoscopic dilation or surgery”
✓ “De-escalation of anti-TNF treatment (either reducing the dose or increasing the administration interval) in IBD is generally recommendable”
**EXTRAINTESTINAL MANIFESTATIONS**
✓ “In hospitalized UC, thromboprophylaxis is not indicated, as they are usually young (and therefore at low risk) and have rectal bleeding (which could worsen with anticoagulation)”
✓ “Ocular manifestations of IBD are never an emergency, and therefore, patients experiencing them should be referred to the ophthalmologist for deferred, outpatient evaluation”
**ANEMIA**
✓ “Anemia (i.e., low hemoglobin levels), but not iron deficiency (i.e., low ferritin levels), is the only significant laboratory finding”
✓ “The impact of anemia on the quality of life of patients with IBD is quite limited”
✓ “Since mild anemia is common in patients with IBD, and its clinical impact is only evident when the anemia is severe, iron therapy is rarely necessary”
✓ “When administering oral iron treatment, higher-than-usual doses should be used because its absorption is often decreased in patients with IBD”
✓ “In patients with IBD, intravenous iron administration should be reserved for cases of severe anemia (e.g., hemoglobin < 8 g/dL)”
**ELDERLY PATIENTS**
✓ “In elderly patients with IBD, the use of biological drugs should be avoided at all costs”
**PREGNANCY**
✓ “During pregnancy, endoscopic examinations should not be performed even if they are clearly indicated, due to the risk of harming the fetus”
✓ “In pregnant women, due to the risk that the medications pose to the fetus, efforts should be made to administer the minimum possible treatment for IBD, even if it means that some intestinal activity persists”
✓ “Biological agents are not safe during pregnancy, and therefore, they should be discontinued before the third trimester”
✓ “Breastfeeding is contraindicated while the mother is undergoing treatment with biological agents”
✓ “In children exposed in utero to biologics, non-live inactivated vaccines are less effective and safe”
✓ “In children exposed in utero to biologics, all live-attenuated vaccines are safe”
✓ “Administration of a live-attenuated vaccine to a breastfed infant while the mother is receiving anti-TNF agents is not recommended unless infant anti-TNF serum levels are undetectable”
**SURGERY**
✓ “In CD, surgery always represents the failure of medicine and is only indicated when medical treatments fail”
✓ “In patients with acute severe UC, surgery should be delayed as much as possible”
✓ “Most drugs used in IBD treatment (corticosteroids, thiopurines, and biologics) equally increase the risk of postoperative complications”
✓ “Previous failure with an anti-TNF agent does necessarily warrant switching to a drug with a different mechanism of action (such as vedolizumab or ustekinumab) to prevent post-operative recurrence of CD”

**ABBREVIATIONS:** anti-tumor necrosis factor (anti-TNF), cytomegalovirus (CMV), Crohn’s disease (CD), inflammatory bowel disease (IBD), ulcerative colitis (UC).

## Data Availability

No new data were generated.

## Abbreviations

Anti-tumor necrosis factor (anti-TNF), Bacillus Calmette–Guérin (BCG), European Crohn’s and Colitis Organisation (ECCO), Clostridioides difficile (C. difficile), cytomegalovirus (CMV), Crohn’s disease (CD), direct healthcare professional communication (DHPC), Epstein–Barr virus (EBV), European Medicines Agency (EMA), hepatitis A virus (HAV), hepatitis B virus (HBV), hepatitis C virus (HCV), inflammatory bowel disease (IBD), post-operative recurrence (POR), ulcerative colitis (UC).
